# Supplementary material for: Not all West Nile virus lineages behave alike: dose-response differences between lineages 1 and 2
Source: Sci Rep. 2026 Aug 4;16:24001. doi: 10.1038/s41598-026-64742-y (PMC13438650; doi:10.1038/s41598-026-64742-y)
Supplement: Supplementary file 2 [file 41598_2026_64742_MOESM2_ESM.docx]

Supplementary Table 2: All analyzed mosquitoes with their corresponding infection status, transmission status, and body titer. Experiments were conducted at an incubation temperature of 24±5°C.

| **Mosquito species** | **WNV Lineage** | **Blood meal titer in TCID50/mL** | **Temperature in °C** | **Incubation period in days post exposure** | **Result infection** | **Result transmission** | **Body titer in viral RNA copies / mosquito body** |
| --- | --- | --- | --- | --- | --- | --- | --- |
| Aedes albopictus | 2 | 1.00E+07 | 24 | 14 | Neg | Neg | 0.00E+00 |
| Aedes albopictus | 2 | 1.00E+07 | 24 | 14 | Neg | Neg | 0.00E+00 |
| Aedes albopictus | 1 | 1.00E+06 | 24 | 14 | Pos | Neg | 1.02E+02 |
| Aedes albopictus | 1 | 1.00E+05 | 24 | 14 | Pos | Neg | 1.11E+02 |
| Aedes albopictus | 1 | 1.00E+06 | 24 | 14 | Pos | Neg | 1.13E+02 |
| Aedes albopictus | 2 | 1.00E+06 | 24 | 14 | Pos | Neg | 1.26E+02 |
| Culex torrentium | 2 | 1.00E+05 | 24 | 14 | Pos | Neg | 1.39E+02 |
| Aedes albopictus | 2 | 1.00E+07 | 24 | 14 | Neg | Neg | 0.00E+00 |
| Aedes albopictus | 1 | 1.00E+05 | 24 | 14 | Pos | Neg | 1.40E+02 |
| Aedes albopictus | 2 | 1.00E+07 | 24 | 14 | Neg | Neg | 0.00E+00 |
| Aedes albopictus | 2 | 1.00E+06 | 24 | 14 | Pos | Neg | 1.69E+02 |
| Culex torrentium | 2 | 1.00E+07 | 24 | 14 | Pos | Neg | 1.75E+02 |
| Aedes albopictus | 1 | 1.00E+06 | 24 | 14 | Pos | Neg | 2.15E+02 |
| Aedes albopictus | 2 | 1.00E+05 | 24 | 14 | Neg | Neg | 0.00E+00 |
| Aedes albopictus | 2 | 1.00E+05 | 24 | 14 | Neg | Neg | 0.00E+00 |
| Aedes albopictus | 2 | 1.00E+05 | 24 | 14 | Neg | Neg | 0.00E+00 |
| Aedes albopictus | 2 | 1.00E+05 | 24 | 14 | Neg | Neg | 0.00E+00 |
| Aedes albopictus | 2 | 1.00E+05 | 24 | 14 | Neg | Neg | 0.00E+00 |
| Aedes albopictus | 2 | 1.00E+05 | 24 | 14 | Neg | Neg | 0.00E+00 |
| Aedes albopictus | 2 | 1.00E+05 | 24 | 14 | Neg | Neg | 0.00E+00 |
| Aedes albopictus | 2 | 1.00E+05 | 24 | 14 | Neg | Neg | 0.00E+00 |
| Culex torrentium | 2 | 1.00E+04 | 24 | 14 | Pos | Neg | 2.16E+02 |
| Aedes albopictus | 2 | 1.00E+05 | 24 | 14 | Neg | Neg | 0.00E+00 |
| Aedes albopictus | 2 | 1.00E+06 | 24 | 14 | Pos | Neg | 2.24E+02 |
| Aedes albopictus | 1 | 1.00E+05 | 24 | 14 | Pos | Neg | 2.24E+02 |
| Aedes albopictus | 2 | 1.00E+05 | 24 | 14 | Neg | Neg | 0.00E+00 |
| Aedes albopictus | 2 | 1.00E+05 | 24 | 14 | Neg | Neg | 0.00E+00 |
| Aedes albopictus | 2 | 1.00E+05 | 24 | 14 | Neg | Neg | 0.00E+00 |
| Aedes albopictus | 2 | 1.00E+05 | 24 | 14 | Neg | Neg | 0.00E+00 |
| Aedes albopictus | 2 | 1.00E+05 | 24 | 14 | Neg | Neg | 0.00E+00 |
| Culex pipiens pipiens | 1 | 1.00E+06 | 24 | 14 | Pos | Neg | 2.26E+02 |
| Culex torrentium | 2 | 1.00E+07 | 24 | 14 | Pos | Neg | 2.33E+02 |
| Culex torrentium | 2 | 1.00E+04 | 24 | 14 | Pos | Neg | 2.35E+02 |
| Aedes albopictus | 1 | 1.00E+06 | 24 | 14 | Pos | Neg | 2.59E+02 |
| Aedes albopictus | 2 | 1.00E+05 | 24 | 14 | Neg | Neg | 0.00E+00 |
| Aedes albopictus | 2 | 1.00E+05 | 24 | 14 | Neg | Neg | 0.00E+00 |
| Aedes albopictus | 2 | 1.00E+05 | 24 | 14 | Neg | Neg | 0.00E+00 |
| Culex torrentium | 1 | 1.00E+07 | 24 | 14 | Pos | Neg | 2.61E+02 |
| Aedes albopictus | 2 | 1.00E+05 | 24 | 14 | Neg | Neg | 0.00E+00 |
| Aedes albopictus | 2 | 1.00E+05 | 24 | 14 | Neg | Neg | 0.00E+00 |
| Aedes albopictus | 2 | 1.00E+05 | 24 | 14 | Neg | Neg | 0.00E+00 |
| Aedes albopictus | 2 | 1.00E+05 | 24 | 14 | Neg | Neg | 0.00E+00 |
| Aedes albopictus | 1 | 1.00E+05 | 24 | 14 | Neg | Neg | 0.00E+00 |
| Culex torrentium | 2 | 1.00E+05 | 24 | 14 | Pos | Neg | 2.78E+02 |
| Aedes albopictus | 1 | 1.00E+05 | 24 | 14 | Neg | Neg | 0.00E+00 |
| Aedes albopictus | 1 | 1.00E+05 | 24 | 14 | Neg | Neg | 0.00E+00 |
| Aedes albopictus | 1 | 1.00E+05 | 24 | 14 | Neg | Neg | 0.00E+00 |
| Aedes albopictus | 1 | 1.00E+05 | 24 | 14 | Neg | Neg | 0.00E+00 |
| Aedes albopictus | 1 | 1.00E+05 | 24 | 14 | Neg | Neg | 0.00E+00 |
| Culex torrentium | 2 | 1.00E+04 | 24 | 14 | Pos | Neg | 2.88E+02 |
| Aedes albopictus | 1 | 1.00E+05 | 24 | 14 | Neg | Neg | 0.00E+00 |
| Aedes albopictus | 1 | 1.00E+05 | 24 | 14 | Neg | Neg | 0.00E+00 |
| Aedes albopictus | 2 | 1.00E+06 | 24 | 14 | Pos | Neg | 2.89E+02 |
| Aedes albopictus | 1 | 1.00E+05 | 24 | 14 | Neg | Neg | 0.00E+00 |
| Aedes albopictus | 1 | 1.00E+05 | 24 | 14 | Neg | Neg | 0.00E+00 |
| Aedes albopictus | 1 | 1.00E+05 | 24 | 14 | Neg | Neg | 0.00E+00 |
| Aedes albopictus | 2 | 1.00E+07 | 24 | 14 | Pos | Neg | 2.99E+02 |
| Aedes albopictus | 1 | 1.00E+05 | 24 | 14 | Neg | Neg | 0.00E+00 |
| Aedes albopictus | 1 | 1.00E+05 | 24 | 14 | Neg | Neg | 0.00E+00 |
| Aedes albopictus | 1 | 1.00E+05 | 24 | 14 | Neg | Neg | 0.00E+00 |
| Aedes albopictus | 1 | 1.00E+06 | 24 | 14 | Pos | Neg | 3.08E+02 |
| Aedes albopictus | 2 | 1.00E+05 | 24 | 14 | Pos | Neg | 3.13E+02 |
| Aedes albopictus | 1 | 1.00E+07 | 24 | 14 | Neg | Neg | 0.00E+00 |
| Aedes albopictus | 1 | 1.00E+07 | 24 | 14 | Neg | Neg | 0.00E+00 |
| Aedes albopictus | 1 | 1.00E+07 | 24 | 14 | Neg | Neg | 0.00E+00 |
| Aedes albopictus | 1 | 1.00E+07 | 24 | 14 | Neg | Neg | 0.00E+00 |
| Aedes albopictus | 1 | 1.00E+07 | 24 | 14 | Neg | Neg | 0.00E+00 |
| Aedes albopictus | 1 | 1.00E+07 | 24 | 14 | Neg | Neg | 0.00E+00 |
| Aedes albopictus | 1 | 1.00E+07 | 24 | 14 | Neg | Neg | 0.00E+00 |
| Aedes albopictus | 1 | 1.00E+07 | 24 | 14 | Neg | Neg | 0.00E+00 |
| Aedes albopictus | 1 | 1.00E+06 | 24 | 14 | Pos | Neg | 3.22E+02 |
| Culex pipiens pipiens | 2 | 1.00E+06 | 24 | 14 | Pos | Neg | 3.26E+02 |
| Culex torrentium | 1 | 1.00E+05 | 24 | 14 | Pos | Neg | 3.43E+02 |
| Culex torrentium | 1 | 1.00E+07 | 24 | 14 | Pos | Neg | 3.58E+02 |
| Culex torrentium | 2 | 1.00E+05 | 24 | 14 | Pos | Neg | 3.71E+02 |
| Aedes albopictus | 1 | 1.00E+07 | 24 | 14 | Neg | Neg | 0.00E+00 |
| Aedes albopictus | 1 | 1.00E+07 | 24 | 14 | Neg | Neg | 0.00E+00 |
| Culex pipiens pipiens | 1 | 1.00E+06 | 24 | 14 | Pos | Neg | 3.75E+02 |
| Aedes albopictus | 1 | 1.00E+07 | 24 | 14 | Pos | Neg | 3.76E+02 |
| Culex torrentium | 2 | 1.00E+05 | 24 | 14 | Pos | Neg | 3.76E+02 |
| Aedes albopictus | 1 | 1.00E+07 | 24 | 14 | Neg | Neg | 0.00E+00 |
| Aedes albopictus | 1 | 1.00E+07 | 24 | 14 | Neg | Neg | 0.00E+00 |
| Aedes albopictus | 1 | 1.00E+07 | 24 | 14 | Pos | Neg | 3.88E+02 |
| Culex pipiens pipiens | 1 | 1.00E+07 | 24 | 14 | Pos | Neg | 4.01E+02 |
| Culex torrentium | 2 | 1.00E+04 | 24 | 14 | Pos | Neg | 4.06E+02 |
| Aedes albopictus | 1 | 1.00E+05 | 24 | 14 | Pos | Neg | 4.09E+02 |
| Aedes albopictus | 2 | 1.00E+06 | 24 | 14 | Pos | Neg | 4.24E+02 |
| Culex pipiens pipiens | 2 | 1.00E+07 | 24 | 14 | Pos | Neg | 4.26E+02 |
| Aedes albopictus | 1 | 1.00E+06 | 24 | 14 | Pos | Neg | 4.26E+02 |
| Culex torrentium | 2 | 1.00E+05 | 24 | 14 | Pos | Neg | 4.29E+02 |
| Aedes albopictus | 2 | 1.00E+06 | 24 | 14 | Pos | Neg | 4.36E+02 |
| Aedes albopictus | 1 | 1.00E+05 | 24 | 14 | Pos | Neg | 4.38E+02 |
| Aedes albopictus | 1 | 1.00E+06 | 24 | 14 | Pos | Neg | 4.50E+02 |
| Culex torrentium | 2 | 1.00E+07 | 24 | 14 | Pos | Neg | 4.55E+02 |
| Culex torrentium | 2 | 1.00E+04 | 24 | 14 | Pos | Neg | 4.64E+02 |
| Culex pipiens pipiens | 2 | 1.00E+05 | 24 | 14 | Pos | Neg | 4.68E+02 |
| Aedes albopictus | 1 | 1.00E+06 | 24 | 14 | Pos | Neg | 4.86E+02 |
| Aedes albopictus | 1 | 1.00E+05 | 24 | 14 | Pos | Neg | 5.00E+02 |
| Aedes albopictus | 1 | 1.00E+06 | 24 | 14 | Pos | Neg | 5.13E+02 |
| Aedes albopictus | 1 | 1.00E+06 | 24 | 14 | Neg | Neg | 0.00E+00 |
| Culex torrentium | 1 | 1.00E+07 | 24 | 14 | Pos | Neg | 5.31E+02 |
| Aedes albopictus | 1 | 1.00E+06 | 24 | 14 | Neg | Neg | 0.00E+00 |
| Culex pipiens pipiens | 1 | 1.00E+05 | 24 | 14 | Pos | Neg | 5.36E+02 |
| Culex pipiens pipiens | 2 | 1.00E+05 | 24 | 14 | Pos | Neg | 5.94E+02 |
| Aedes albopictus | 1 | 1.00E+07 | 24 | 14 | Pos | Neg | 6.12E+02 |
| Culex pipiens pipiens | 2 | 1.00E+05 | 24 | 14 | Pos | Neg | 6.12E+02 |
| Aedes albopictus | 1 | 1.00E+05 | 24 | 14 | Neg | Neg | 0.00E+00 |
| Aedes albopictus | 1 | 1.00E+05 | 24 | 14 | Neg | Neg | 0.00E+00 |
| Aedes albopictus | 1 | 1.00E+05 | 24 | 14 | Pos | Neg | 6.35E+02 |
| Culex pipiens pipiens | 1 | 1.00E+07 | 24 | 14 | Pos | Neg | 6.48E+02 |
| Aedes albopictus | 1 | 1.00E+05 | 24 | 14 | Pos | Neg | 6.57E+02 |
| Culex torrentium | 1 | 1.00E+05 | 24 | 14 | Pos | Neg | 6.80E+02 |
| Aedes albopictus | 2 | 1.00E+06 | 24 | 14 | Pos | Neg | 6.89E+02 |
| Aedes albopictus | 1 | 1.00E+07 | 24 | 14 | Pos | Neg | 6.93E+02 |
| Culex torrentium | 1 | 1.00E+07 | 24 | 14 | Pos | Neg | 6.98E+02 |
| Culex torrentium | 2 | 1.00E+05 | 24 | 14 | Pos | Neg | 7.02E+02 |
| Aedes albopictus | 2 | 1.00E+06 | 24 | 14 | Pos | Neg | 7.16E+02 |
| Culex torrentium | 2 | 1.00E+05 | 24 | 14 | Pos | Neg | 7.20E+02 |
| Culex torrentium | 2 | 1.00E+06 | 24 | 14 | Pos | Neg | 7.34E+02 |
| Aedes albopictus | 2 | 1.00E+07 | 24 | 14 | Pos | Pos | 7.65E+02 |
| Culex torrentium | 2 | 1.00E+05 | 24 | 14 | Pos | Neg | 7.79E+02 |
| Culex torrentium | 1 | 1.00E+05 | 24 | 14 | Pos | Neg | 7.97E+02 |
| Aedes albopictus | 2 | 1.00E+06 | 24 | 14 | Neg | Neg | 0.00E+00 |
| Culex torrentium | 2 | 1.00E+06 | 24 | 14 | Pos | Neg | 8.42E+02 |
| Culex pipiens pipiens | 2 | 1.00E+05 | 24 | 14 | Pos | Neg | 8.42E+02 |
| Aedes albopictus | 2 | 1.00E+06 | 24 | 14 | Neg | Neg | 0.00E+00 |
| Aedes albopictus | 2 | 1.00E+06 | 24 | 14 | Neg | Neg | 0.00E+00 |
| Aedes albopictus | 2 | 1.00E+05 | 24 | 14 | Pos | Neg | 9.14E+02 |
| Aedes albopictus | 1 | 1.00E+07 | 24 | 14 | Pos | Neg | 9.59E+02 |
| Culex torrentium | 2 | 1.00E+06 | 24 | 14 | Pos | Neg | 9.77E+02 |
| Culex pipiens pipiens | 1 | 1.00E+07 | 24 | 14 | Pos | Neg | 9.77E+02 |
| Aedes albopictus | 1 | 1.00E+07 | 24 | 14 | Pos | Neg | 1.00E+03 |
| Culex torrentium | 1 | 1.00E+07 | 24 | 14 | Pos | Neg | 1.05E+03 |
| Aedes albopictus | 2 | 1.00E+06 | 24 | 14 | Neg | Neg | 0.00E+00 |
| Culex torrentium | 1 | 1.00E+07 | 24 | 14 | Pos | Neg | 1.05E+03 |
| Culex torrentium | 1 | 1.00E+06 | 24 | 14 | Pos | Neg | 1.06E+03 |
| Aedes albopictus | 2 | 1.00E+06 | 24 | 14 | Pos | Neg | 1.10E+03 |
| Aedes albopictus | 2 | 1.00E+06 | 24 | 14 | Pos | Neg | 1.12E+03 |
| Aedes albopictus | 1 | 1.00E+06 | 24 | 14 | Pos | Neg | 1.13E+03 |
| Aedes albopictus | 2 | 1.00E+06 | 24 | 14 | Neg | Neg | 0.00E+00 |
| Aedes albopictus | 2 | 1.00E+06 | 24 | 14 | Neg | Neg | 0.00E+00 |
| Aedes albopictus | 2 | 1.00E+07 | 24 | 14 | Pos | Neg | 1.15E+03 |
| Culex pipiens pipiens | 2 | 1.00E+06 | 24 | 14 | Pos | Neg | 1.15E+03 |
| Aedes albopictus | 1 | 1.00E+07 | 24 | 14 | Pos | Neg | 1.19E+03 |
| Aedes albopictus | 2 | 1.00E+06 | 24 | 14 | Pos | Neg | 1.20E+03 |
| Culex pipiens pipiens | 2 | 1.00E+07 | 24 | 14 | Pos | Neg | 1.21E+03 |
| Aedes albopictus | 1 | 1.00E+06 | 24 | 14 | Pos | Neg | 1.24E+03 |
| Culex torrentium | 2 | 1.00E+04 | 24 | 14 | Pos | Neg | 1.39E+03 |
| Culex torrentium | 2 | 1.00E+07 | 24 | 14 | Neg | Neg | 0.00E+00 |
| Culex torrentium | 2 | 1.00E+07 | 24 | 14 | Neg | Neg | 0.00E+00 |
| Culex pipiens pipiens | 1 | 1.00E+07 | 24 | 14 | Pos | Neg | 1.40E+03 |
| Aedes albopictus | 2 | 1.00E+06 | 24 | 14 | Pos | Neg | 1.40E+03 |
| Culex torrentium | 2 | 1.00E+05 | 24 | 14 | Pos | Neg | 1.42E+03 |
| Culex pipiens pipiens | 2 | 1.00E+07 | 24 | 14 | Pos | Neg | 1.42E+03 |
| Culex torrentium | 1 | 1.00E+07 | 24 | 14 | Pos | Neg | 1.54E+03 |
| Culex torrentium | 2 | 1.00E+07 | 24 | 14 | Neg | Neg | 0.00E+00 |
| Culex pipiens pipiens | 2 | 1.00E+06 | 24 | 14 | Pos | Neg | 1.56E+03 |
| Culex torrentium | 2 | 1.00E+07 | 24 | 14 | Neg | Neg | 0.00E+00 |
| Culex torrentium | 2 | 1.00E+07 | 24 | 14 | Neg | Neg | 0.00E+00 |
| Aedes albopictus | 1 | 1.00E+05 | 24 | 14 | Pos | Neg | 1.60E+03 |
| Culex pipiens pipiens | 1 | 1.00E+06 | 24 | 14 | Pos | Neg | 1.65E+03 |
| Culex torrentium | 2 | 1.00E+07 | 24 | 14 | Neg | Neg | 0.00E+00 |
| Culex pipiens pipiens | 1 | 1.00E+05 | 24 | 14 | Pos | Neg | 1.68E+03 |
| Culex pipiens pipiens | 2 | 1.00E+07 | 24 | 14 | Neg | Neg | 0.00E+00 |
| Culex pipiens pipiens | 2 | 1.00E+07 | 24 | 14 | Neg | Neg | 0.00E+00 |
| Culex pipiens pipiens | 2 | 1.00E+07 | 24 | 14 | Neg | Neg | 0.00E+00 |
| Culex pipiens pipiens | 2 | 1.00E+07 | 24 | 14 | Neg | Neg | 0.00E+00 |
| Culex pipiens pipiens | 2 | 1.00E+07 | 24 | 14 | Neg | Neg | 0.00E+00 |
| Culex pipiens pipiens | 2 | 1.00E+07 | 24 | 14 | Neg | Neg | 0.00E+00 |
| Aedes albopictus | 2 | 1.00E+07 | 24 | 14 | Pos | Neg | 1.69E+03 |
| Aedes albopictus | 1 | 1.00E+07 | 24 | 14 | Pos | Neg | 1.71E+03 |
| Culex pipiens pipiens | 2 | 1.00E+07 | 24 | 14 | Neg | Neg | 0.00E+00 |
| Aedes albopictus | 2 | 1.00E+07 | 24 | 14 | Pos | Neg | 1.72E+03 |
| Aedes albopictus | 1 | 1.00E+06 | 24 | 14 | Neg | Neg | 0.00E+00 |
| Aedes albopictus | 1 | 1.00E+06 | 24 | 14 | Neg | Neg | 0.00E+00 |
| Aedes albopictus | 1 | 1.00E+06 | 24 | 14 | Neg | Neg | 0.00E+00 |
| Aedes albopictus | 1 | 1.00E+06 | 24 | 14 | Neg | Neg | 0.00E+00 |
| Culex pipiens pipiens | 2 | 1.00E+06 | 24 | 14 | Pos | Neg | 1.81E+03 |
| Aedes albopictus | 1 | 1.00E+06 | 24 | 14 | Neg | Neg | 0.00E+00 |
| Aedes albopictus | 1 | 1.00E+06 | 24 | 14 | Neg | Neg | 0.00E+00 |
| Aedes albopictus | 1 | 1.00E+07 | 24 | 14 | Pos | Neg | 1.88E+03 |
| Culex torrentium | 1 | 1.00E+07 | 24 | 14 | Pos | Neg | 1.93E+03 |
| Culex torrentium | 1 | 1.00E+05 | 24 | 14 | Pos | Neg | 1.98E+03 |
| Culex pipiens pipiens | 2 | 1.00E+07 | 24 | 14 | Neg | Neg | 0.00E+00 |
| Culex pipiens pipiens | 2 | 1.00E+07 | 24 | 14 | Pos | Neg | 2.05E+03 |
| Culex torrentium | 1 | 1.00E+06 | 24 | 14 | Pos | Neg | 2.08E+03 |
| Culex pipiens pipiens | 1 | 1.00E+05 | 24 | 14 | Pos | Neg | 2.08E+03 |
| Culex pipiens pipiens | 2 | 1.00E+06 | 24 | 14 | Pos | Neg | 2.12E+03 |
| Aedes albopictus | 2 | 1.00E+05 | 24 | 14 | Pos | Neg | 2.18E+03 |
| Culex pipiens pipiens | 2 | 1.00E+06 | 24 | 14 | Pos | Neg | 2.19E+03 |
| Aedes albopictus | 1 | 1.00E+07 | 24 | 14 | Pos | Neg | 2.21E+03 |
| Culex torrentium | 2 | 1.00E+06 | 24 | 14 | Pos | Neg | 2.57E+03 |
| Culex pipiens pipiens | 1 | 1.00E+06 | 24 | 14 | Pos | Neg | 2.62E+03 |
| Culex pipiens pipiens | 1 | 1.00E+06 | 24 | 14 | Pos | Neg | 2.63E+03 |
| Culex torrentium | 2 | 1.00E+06 | 24 | 14 | Neg | Neg | 0.00E+00 |
| Culex torrentium | 2 | 1.00E+06 | 24 | 14 | Neg | Neg | 0.00E+00 |
| Culex torrentium | 2 | 1.00E+06 | 24 | 14 | Neg | Neg | 0.00E+00 |
| Culex torrentium | 2 | 1.00E+06 | 24 | 14 | Neg | Neg | 0.00E+00 |
| Culex torrentium | 2 | 1.00E+06 | 24 | 14 | Neg | Neg | 0.00E+00 |
| Culex torrentium | 2 | 1.00E+07 | 24 | 14 | Pos | Neg | 2.70E+03 |
| Culex torrentium | 2 | 1.00E+06 | 24 | 14 | Neg | Neg | 0.00E+00 |
| Culex pipiens pipiens | 1 | 1.00E+07 | 24 | 14 | Pos | Neg | 2.88E+03 |
| Culex pipiens pipiens | 2 | 1.00E+06 | 24 | 14 | Neg | Neg | 0.00E+00 |
| Culex pipiens pipiens | 2 | 1.00E+06 | 24 | 14 | Neg | Neg | 0.00E+00 |
| Culex torrentium | 1 | 1.00E+07 | 24 | 14 | Pos | Neg | 2.89E+03 |
| Aedes albopictus | 2 | 1.00E+06 | 24 | 14 | Pos | Neg | 2.90E+03 |
| Culex torrentium | 2 | 1.00E+06 | 24 | 14 | Neg | Neg | 0.00E+00 |
| Aedes albopictus | 2 | 1.00E+06 | 24 | 14 | Pos | Neg | 2.95E+03 |
| Culex torrentium | 2 | 1.00E+06 | 24 | 14 | Neg | Neg | 0.00E+00 |
| Aedes albopictus | 2 | 1.00E+05 | 24 | 14 | Pos | Neg | 3.14E+03 |
| Culex torrentium | 1 | 1.00E+07 | 24 | 14 | Pos | Neg | 3.16E+03 |
| Culex pipiens pipiens | 2 | 1.00E+06 | 24 | 14 | Neg | Neg | 0.00E+00 |
| Culex pipiens pipiens | 2 | 1.00E+06 | 24 | 14 | Neg | Neg | 0.00E+00 |
| Culex pipiens pipiens | 2 | 1.00E+06 | 24 | 14 | Neg | Neg | 0.00E+00 |
| Aedes albopictus | 1 | 1.00E+05 | 24 | 14 | Pos | Neg | 3.24E+03 |
| Culex pipiens pipiens | 2 | 1.00E+06 | 24 | 14 | Neg | Neg | 0.00E+00 |
| Culex pipiens pipiens | 2 | 1.00E+06 | 24 | 14 | Pos | Neg | 3.66E+03 |
| Aedes albopictus | 2 | 1.00E+05 | 24 | 14 | Pos | Neg | 3.73E+03 |
| Culex pipiens pipiens | 2 | 1.00E+06 | 24 | 14 | Neg | Neg | 0.00E+00 |
| Culex torrentium | 2 | 1.00E+06 | 24 | 14 | Pos | Neg | 4.20E+03 |
| Aedes albopictus | 2 | 1.00E+06 | 24 | 14 | Pos | Neg | 4.24E+03 |
| Aedes albopictus | 2 | 1.00E+07 | 24 | 14 | Pos | Neg | 4.48E+03 |
| Culex pipiens pipiens | 2 | 1.00E+06 | 24 | 14 | Pos | Neg | 4.53E+03 |
| Culex pipiens pipiens | 2 | 1.00E+06 | 24 | 14 | Pos | Neg | 4.58E+03 |
| Culex pipiens pipiens | 2 | 1.00E+06 | 24 | 14 | Neg | Neg | 0.00E+00 |
| Culex pipiens pipiens | 2 | 1.00E+06 | 24 | 14 | Neg | Neg | 0.00E+00 |
| Culex torrentium | 2 | 1.00E+06 | 24 | 14 | Pos | Neg | 5.08E+03 |
| Culex torrentium | 2 | 1.00E+06 | 24 | 14 | Pos | Neg | 5.90E+03 |
| Culex pipiens pipiens | 2 | 1.00E+06 | 24 | 14 | Neg | Neg | 0.00E+00 |
| Culex pipiens pipiens | 2 | 1.00E+06 | 24 | 14 | Neg | Neg | 0.00E+00 |
| Aedes albopictus | 2 | 1.00E+07 | 24 | 14 | Pos | Neg | 6.12E+03 |
| Culex torrentium | 1 | 1.00E+07 | 24 | 14 | Pos | Neg | 6.17E+03 |
| Aedes albopictus | 2 | 1.00E+06 | 24 | 14 | Pos | Neg | 6.30E+03 |
| Aedes albopictus | 1 | 1.00E+07 | 24 | 14 | Pos | Neg | 6.44E+03 |
| Aedes albopictus | 2 | 1.00E+05 | 24 | 14 | Pos | Neg | 6.80E+03 |
| Culex torrentium | 2 | 1.00E+06 | 24 | 14 | Pos | Neg | 7.96E+03 |
| Culex torrentium | 2 | 1.00E+07 | 24 | 14 | Neg | Neg | 0.00E+00 |
| Culex torrentium | 2 | 1.00E+06 | 24 | 14 | Pos | Neg | 8.06E+03 |
| Aedes albopictus | 2 | 1.00E+07 | 24 | 14 | Pos | Neg | 8.73E+03 |
| Aedes albopictus | 2 | 1.00E+07 | 24 | 14 | Pos | Neg | 8.87E+03 |
| Aedes albopictus | 2 | 1.00E+07 | 24 | 14 | Pos | Neg | 9.99E+03 |
| Culex pipiens pipiens | 2 | 1.00E+07 | 24 | 14 | Neg | Neg | 0.00E+00 |
| Culex pipiens pipiens | 2 | 1.00E+07 | 24 | 14 | Neg | Neg | 0.00E+00 |
| Culex torrentium | 2 | 1.00E+07 | 24 | 14 | Pos | Neg | 1.09E+04 |
| Culex pipiens pipiens | 2 | 1.00E+07 | 24 | 14 | Neg | Neg | 0.00E+00 |
| Culex pipiens pipiens | 2 | 1.00E+07 | 24 | 14 | Neg | Neg | 0.00E+00 |
| Culex pipiens pipiens | 1 | 1.00E+07 | 24 | 14 | Pos | Neg | 1.12E+04 |
| Culex pipiens pipiens | 2 | 1.00E+06 | 24 | 14 | Neg | Neg | 0.00E+00 |
| Culex pipiens pipiens | 2 | 1.00E+06 | 24 | 14 | Neg | Neg | 0.00E+00 |
| Culex pipiens pipiens | 2 | 1.00E+06 | 24 | 14 | Pos | Neg | 1.17E+04 |
| Aedes albopictus | 1 | 1.00E+07 | 24 | 14 | Pos | Neg | 1.42E+04 |
| Culex pipiens pipiens | 2 | 1.00E+06 | 24 | 14 | Neg | Neg | 0.00E+00 |
| Culex pipiens pipiens | 2 | 1.00E+06 | 24 | 14 | Pos | Neg | 1.49E+04 |
| Culex pipiens pipiens | 2 | 1.00E+06 | 24 | 14 | Neg | Neg | 0.00E+00 |
| Culex torrentium | 2 | 1.00E+06 | 24 | 14 | Neg | Neg | 0.00E+00 |
| Aedes albopictus | 2 | 1.00E+07 | 24 | 14 | Pos | Neg | 1.53E+04 |
| Aedes albopictus | 2 | 1.00E+07 | 24 | 14 | Pos | Pos | 1.62E+04 |
| Aedes albopictus | 2 | 1.00E+07 | 24 | 14 | Pos | Pos | 1.73E+04 |
| Culex torrentium | 2 | 1.00E+06 | 24 | 14 | Neg | Neg | 0.00E+00 |
| Culex pipiens pipiens | 2 | 1.00E+07 | 24 | 14 | Pos | Neg | 1.86E+04 |
| Aedes albopictus | 2 | 1.00E+05 | 24 | 14 | Pos | Neg | 1.88E+04 |
| Aedes albopictus | 2 | 1.00E+07 | 24 | 14 | Pos | Neg | 2.18E+04 |
| Aedes albopictus | 2 | 1.00E+07 | 24 | 14 | Pos | Neg | 2.25E+04 |
| Culex pipiens pipiens | 2 | 1.00E+07 | 24 | 14 | Pos | Neg | 2.34E+04 |
| Culex torrentium | 2 | 1.00E+06 | 24 | 14 | Pos | Neg | 2.37E+04 |
| Aedes albopictus | 2 | 1.00E+07 | 24 | 14 | Pos | Pos | 2.51E+04 |
| Aedes albopictus | 2 | 1.00E+06 | 24 | 14 | Pos | Neg | 2.81E+04 |
| Culex pipiens pipiens | 2 | 1.00E+06 | 24 | 14 | Pos | Neg | 3.21E+04 |
| Culex torrentium | 2 | 1.00E+06 | 24 | 14 | Pos | Neg | 4.13E+04 |
| Aedes albopictus | 2 | 1.00E+07 | 24 | 14 | Pos | Neg | 4.32E+04 |
| Culex torrentium | 2 | 1.00E+06 | 24 | 14 | Pos | Neg | 4.64E+04 |
| Aedes albopictus | 1 | 1.00E+07 | 24 | 14 | Pos | Neg | 4.82E+04 |
| Culex pipiens pipiens | 2 | 1.00E+06 | 24 | 14 | Pos | Neg | 4.98E+04 |
| Aedes albopictus | 2 | 1.00E+07 | 24 | 14 | Pos | Neg | 5.58E+04 |
| Culex pipiens pipiens | 2 | 1.00E+07 | 24 | 14 | Pos | Neg | 6.78E+04 |
| Culex torrentium | 2 | 1.00E+06 | 24 | 14 | Pos | Neg | 6.98E+04 |
| Aedes albopictus | 2 | 1.00E+06 | 24 | 14 | Pos | Neg | 8.46E+04 |
| Culex torrentium | 2 | 1.00E+05 | 24 | 14 | Neg | Neg | 0.00E+00 |
| Culex torrentium | 2 | 1.00E+05 | 24 | 14 | Neg | Neg | 0.00E+00 |
| Culex torrentium | 2 | 1.00E+05 | 24 | 14 | Neg | Neg | 0.00E+00 |
| Culex torrentium | 2 | 1.00E+05 | 24 | 14 | Neg | Neg | 0.00E+00 |
| Culex torrentium | 2 | 1.00E+05 | 24 | 14 | Neg | Neg | 0.00E+00 |
| Aedes albopictus | 1 | 1.00E+06 | 24 | 14 | Pos | Neg | 8.91E+04 |
| Culex torrentium | 2 | 1.00E+06 | 24 | 14 | Pos | Neg | 9.05E+04 |
| Aedes albopictus | 2 | 1.00E+07 | 24 | 14 | Pos | Neg | 1.00E+05 |
| Culex torrentium | 2 | 1.00E+05 | 24 | 14 | Neg | Neg | 0.00E+00 |
| Culex torrentium | 2 | 1.00E+05 | 24 | 14 | Neg | Neg | 0.00E+00 |
| Culex torrentium | 2 | 1.00E+05 | 24 | 14 | Neg | Neg | 0.00E+00 |
| Culex torrentium | 2 | 1.00E+05 | 24 | 14 | Neg | Neg | 0.00E+00 |
| Culex torrentium | 2 | 1.00E+05 | 24 | 14 | Neg | Neg | 0.00E+00 |
| Aedes albopictus | 1 | 1.00E+05 | 24 | 14 | Pos | Neg | 1.37E+05 |
| Culex torrentium | 2 | 1.00E+05 | 24 | 14 | Neg | Neg | 0.00E+00 |
| Culex torrentium | 2 | 1.00E+05 | 24 | 14 | Neg | Neg | 0.00E+00 |
| Culex torrentium | 2 | 1.00E+05 | 24 | 14 | Neg | Neg | 0.00E+00 |
| Culex torrentium | 2 | 1.00E+05 | 24 | 14 | Neg | Neg | 0.00E+00 |
| Culex torrentium | 2 | 1.00E+05 | 24 | 14 | Neg | Neg | 0.00E+00 |
| Aedes albopictus | 2 | 1.00E+07 | 24 | 14 | Pos | Pos | 1.61E+05 |
| Culex torrentium | 2 | 1.00E+05 | 24 | 14 | Neg | Neg | 0.00E+00 |
| Culex torrentium | 2 | 1.00E+06 | 24 | 14 | Pos | Neg | 2.01E+05 |
| Aedes albopictus | 1 | 1.00E+06 | 24 | 14 | Pos | Neg | 5.33E+05 |
| Culex torrentium | 2 | 1.00E+05 | 24 | 14 | Neg | Neg | 0.00E+00 |
| Culex torrentium | 1 | 1.00E+07 | 24 | 14 | Pos | Neg | 1.40E+06 |
| Culex torrentium | 2 | 1.00E+04 | 24 | 14 | Neg | Neg | 0.00E+00 |
| Culex torrentium | 2 | 1.00E+04 | 24 | 14 | Neg | Neg | 0.00E+00 |
| Culex torrentium | 1 | 1.00E+07 | 24 | 14 | Pos | Neg | 2.55E+06 |
| Culex torrentium | 2 | 1.00E+04 | 24 | 14 | Neg | Neg | 0.00E+00 |
| Culex torrentium | 2 | 1.00E+04 | 24 | 14 | Neg | Neg | 0.00E+00 |
| Culex torrentium | 2 | 1.00E+04 | 24 | 14 | Neg | Neg | 0.00E+00 |
| Culex torrentium | 2 | 1.00E+04 | 24 | 14 | Neg | Neg | 0.00E+00 |
| Culex torrentium | 2 | 1.00E+04 | 24 | 14 | Neg | Neg | 0.00E+00 |
| Culex torrentium | 2 | 1.00E+04 | 24 | 14 | Neg | Neg | 0.00E+00 |
| Culex torrentium | 2 | 1.00E+04 | 24 | 14 | Neg | Neg | 0.00E+00 |
| Culex torrentium | 2 | 1.00E+04 | 24 | 14 | Neg | Neg | 0.00E+00 |
| Culex torrentium | 2 | 1.00E+04 | 24 | 14 | Neg | Neg | 0.00E+00 |
| Culex torrentium | 2 | 1.00E+04 | 24 | 14 | Neg | Neg | 0.00E+00 |
| Culex pipiens pipiens | 1 | 1.00E+07 | 24 | 14 | Neg | Neg | 0.00E+00 |
| Culex torrentium | 2 | 1.00E+07 | 24 | 14 | Pos | Neg | 2.75E+06 |
| Culex torrentium | 2 | 1.00E+07 | 24 | 14 | Pos | Neg | 3.63E+06 |
| Culex pipiens pipiens | 1 | 1.00E+07 | 24 | 14 | Neg | Neg | 0.00E+00 |
| Culex pipiens pipiens | 1 | 1.00E+07 | 24 | 14 | Neg | Neg | 0.00E+00 |
| Culex torrentium | 2 | 1.00E+07 | 24 | 14 | Pos | Neg | 7.20E+06 |
| Culex pipiens pipiens | 1 | 1.00E+07 | 24 | 14 | Neg | Neg | 0.00E+00 |
| Culex pipiens pipiens | 1 | 1.00E+07 | 24 | 14 | Neg | Neg | 0.00E+00 |
| Culex torrentium | 1 | 1.00E+07 | 24 | 14 | Pos | Neg | 7.79E+06 |
| Aedes albopictus | 2 | 1.00E+07 | 24 | 14 | Pos | Neg | 8.24E+06 |
| Culex torrentium | 2 | 1.00E+04 | 24 | 14 | Neg | Neg | 0.00E+00 |
| Culex torrentium | 2 | 1.00E+07 | 24 | 14 | Pos | Neg | 9.95E+06 |
| Culex pipiens pipiens | 1 | 1.00E+07 | 24 | 14 | Pos | Neg | 1.14E+07 |
| Culex torrentium | 2 | 1.00E+04 | 24 | 14 | Neg | Neg | 0.00E+00 |
| Culex torrentium | 2 | 1.00E+04 | 24 | 14 | Neg | Neg | 0.00E+00 |
| Culex torrentium | 2 | 1.00E+04 | 24 | 14 | Neg | Neg | 0.00E+00 |
| Culex torrentium | 2 | 1.00E+04 | 24 | 14 | Neg | Neg | 0.00E+00 |
| Culex torrentium | 2 | 1.00E+04 | 24 | 14 | Neg | Neg | 0.00E+00 |
| Culex pipiens pipiens | 1 | 1.00E+07 | 24 | 14 | Pos | Pos | 1.30E+07 |
| Culex torrentium | 2 | 1.00E+04 | 24 | 14 | Neg | Neg | 0.00E+00 |
| Culex torrentium | 2 | 1.00E+04 | 24 | 14 | Neg | Neg | 0.00E+00 |
| Culex torrentium | 2 | 1.00E+04 | 24 | 14 | Neg | Neg | 0.00E+00 |
| Culex torrentium | 2 | 1.00E+04 | 24 | 14 | Neg | Neg | 0.00E+00 |
| Culex torrentium | 2 | 1.00E+04 | 24 | 14 | Neg | Neg | 0.00E+00 |
| Culex torrentium | 2 | 1.00E+04 | 24 | 14 | Neg | Neg | 0.00E+00 |
| Culex torrentium | 1 | 1.00E+06 | 24 | 14 | Pos | Neg | 1.44E+07 |
| Culex pipiens pipiens | 1 | 1.00E+07 | 24 | 14 | Neg | Neg | 0.00E+00 |
| Culex pipiens pipiens | 1 | 1.00E+07 | 24 | 14 | Neg | Neg | 0.00E+00 |
| Culex torrentium | 1 | 1.00E+07 | 24 | 14 | Pos | Neg | 1.63E+07 |
| Culex torrentium | 2 | 1.00E+07 | 24 | 14 | Pos | Pos | 2.29E+07 |
| Culex pipiens pipiens | 2 | 1.00E+06 | 24 | 14 | Pos | Neg | 2.64E+07 |
| Culex torrentium | 2 | 1.00E+07 | 24 | 14 | Pos | Pos | 2.70E+07 |
| Culex torrentium | 2 | 1.00E+06 | 24 | 14 | Pos | Neg | 3.21E+07 |
| Culex torrentium | 2 | 1.00E+06 | 24 | 14 | Pos | Neg | 3.58E+07 |
| Culex torrentium | 1 | 1.00E+07 | 24 | 14 | Pos | Pos | 3.92E+07 |
| Culex torrentium | 2 | 1.00E+07 | 24 | 14 | Pos | Neg | 4.00E+07 |
| Culex pipiens pipiens | 2 | 1.00E+05 | 24 | 14 | Pos | Neg | 4.91E+07 |
| Culex torrentium | 1 | 1.00E+07 | 24 | 14 | Pos | Neg | 5.36E+07 |
| Culex pipiens pipiens | 2 | 1.00E+07 | 24 | 14 | Pos | Neg | 5.90E+07 |
| Culex torrentium | 2 | 1.00E+07 | 24 | 14 | Pos | Pos | 6.53E+07 |
| Culex torrentium | 1 | 1.00E+07 | 24 | 14 | Neg | Neg | 0.00E+00 |
| Culex torrentium | 1 | 1.00E+07 | 24 | 14 | Neg | Neg | 0.00E+00 |
| Culex torrentium | 1 | 1.00E+07 | 24 | 14 | Pos | Neg | 7.25E+07 |
| Culex torrentium | 2 | 1.00E+06 | 24 | 14 | Pos | Pos | 8.06E+07 |
| Culex torrentium | 2 | 1.00E+07 | 24 | 14 | Pos | Neg | 8.60E+07 |
| Culex torrentium | 1 | 1.00E+06 | 24 | 14 | Neg | Neg | 0.00E+00 |
| Culex torrentium | 1 | 1.00E+06 | 24 | 14 | Neg | Neg | 0.00E+00 |
| Culex torrentium | 1 | 1.00E+06 | 24 | 14 | Neg | Neg | 0.00E+00 |
| Culex torrentium | 1 | 1.00E+06 | 24 | 14 | Neg | Neg | 0.00E+00 |
| Culex torrentium | 1 | 1.00E+06 | 24 | 14 | Neg | Neg | 0.00E+00 |
| Culex torrentium | 1 | 1.00E+06 | 24 | 14 | Neg | Neg | 0.00E+00 |
| Culex torrentium | 1 | 1.00E+06 | 24 | 14 | Neg | Neg | 0.00E+00 |
| Culex torrentium | 1 | 1.00E+06 | 24 | 14 | Neg | Neg | 0.00E+00 |
| Culex torrentium | 1 | 1.00E+06 | 24 | 14 | Neg | Neg | 0.00E+00 |
| Culex torrentium | 1 | 1.00E+06 | 24 | 14 | Neg | Neg | 0.00E+00 |
| Culex torrentium | 1 | 1.00E+06 | 24 | 14 | Neg | Neg | 0.00E+00 |
| Culex pipiens pipiens | 2 | 1.00E+07 | 24 | 14 | Pos | Neg | 8.80E+07 |
| Culex torrentium | 1 | 1.00E+06 | 24 | 14 | Neg | Neg | 0.00E+00 |
| Culex pipiens pipiens | 2 | 1.00E+07 | 24 | 14 | Pos | Neg | 1.17E+08 |
| Culex torrentium | 1 | 1.00E+05 | 24 | 14 | Neg | Neg | 0.00E+00 |
| Culex torrentium | 1 | 1.00E+05 | 24 | 14 | Neg | Neg | 0.00E+00 |
| Culex torrentium | 1 | 1.00E+05 | 24 | 14 | Neg | Neg | 0.00E+00 |
| Culex torrentium | 1 | 1.00E+05 | 24 | 14 | Neg | Neg | 0.00E+00 |
| Culex torrentium | 1 | 1.00E+05 | 24 | 14 | Neg | Neg | 0.00E+00 |
| Culex torrentium | 1 | 1.00E+05 | 24 | 14 | Neg | Neg | 0.00E+00 |
| Culex pipiens pipiens | 2 | 1.00E+07 | 24 | 14 | Pos | Pos | 1.18E+08 |
| Culex pipiens pipiens | 2 | 1.00E+07 | 24 | 14 | Pos | Neg | 1.26E+08 |
| Culex torrentium | 1 | 1.00E+05 | 24 | 14 | Neg | Neg | 0.00E+00 |
| Culex torrentium | 1 | 1.00E+05 | 24 | 14 | Neg | Neg | 0.00E+00 |
| Culex torrentium | 1 | 1.00E+05 | 24 | 14 | Neg | Neg | 0.00E+00 |
| Culex torrentium | 1 | 1.00E+05 | 24 | 14 | Neg | Neg | 0.00E+00 |
| Culex torrentium | 1 | 1.00E+05 | 24 | 14 | Neg | Neg | 0.00E+00 |
| Culex torrentium | 1 | 1.00E+05 | 24 | 14 | Neg | Neg | 0.00E+00 |
| Culex torrentium | 1 | 1.00E+05 | 24 | 14 | Neg | Neg | 0.00E+00 |
| Culex torrentium | 1 | 1.00E+07 | 24 | 14 | Neg | Neg | 0.00E+00 |
| Culex torrentium | 1 | 1.00E+07 | 24 | 14 | Neg | Neg | 0.00E+00 |
| Culex torrentium | 2 | 1.00E+07 | 24 | 14 | Pos | Pos | 1.43E+08 |
| Culex torrentium | 2 | 1.00E+07 | 24 | 14 | Pos | Neg | 1.51E+08 |
| Culex pipiens pipiens | 2 | 1.00E+06 | 24 | 14 | Pos | Neg | 1.54E+08 |
| Culex torrentium | 1 | 1.00E+07 | 24 | 14 | Neg | Neg | 0.00E+00 |
| Culex torrentium | 1 | 1.00E+07 | 24 | 14 | Neg | Neg | 0.00E+00 |
| Culex torrentium | 1 | 1.00E+07 | 24 | 14 | Neg | Neg | 0.00E+00 |
| Culex pipiens pipiens | 1 | 1.00E+07 | 24 | 14 | Pos | Neg | 1.59E+08 |
| Aedes albopictus | 2 | 1.00E+07 | 24 | 14 | Pos | Pos | 1.83E+08 |
| Culex torrentium | 1 | 1.00E+07 | 24 | 14 | Neg | Neg | 0.00E+00 |
| Culex pipiens pipiens | 2 | 1.00E+07 | 24 | 14 | Pos | Neg | 1.95E+08 |
| Culex torrentium | 1 | 1.00E+07 | 24 | 14 | Neg | Neg | 0.00E+00 |
| Culex torrentium | 1 | 1.00E+07 | 24 | 14 | Neg | Neg | 0.00E+00 |
| Culex torrentium | 1 | 1.00E+07 | 24 | 14 | Neg | Neg | 0.00E+00 |
| Culex torrentium | 1 | 1.00E+06 | 24 | 14 | Neg | Neg | 0.00E+00 |
| Culex torrentium | 1 | 1.00E+06 | 24 | 14 | Neg | Neg | 0.00E+00 |
| Culex torrentium | 1 | 1.00E+06 | 24 | 14 | Neg | Neg | 0.00E+00 |
| Culex torrentium | 1 | 1.00E+06 | 24 | 14 | Neg | Neg | 0.00E+00 |
| Culex torrentium | 1 | 1.00E+06 | 24 | 14 | Neg | Neg | 0.00E+00 |
| Culex torrentium | 1 | 1.00E+06 | 24 | 14 | Neg | Neg | 0.00E+00 |
| Culex torrentium | 1 | 1.00E+06 | 24 | 14 | Neg | Neg | 0.00E+00 |
| Culex torrentium | 1 | 1.00E+06 | 24 | 14 | Neg | Neg | 0.00E+00 |
| Culex torrentium | 1 | 1.00E+06 | 24 | 14 | Neg | Neg | 0.00E+00 |
| Culex torrentium | 1 | 1.00E+06 | 24 | 14 | Neg | Neg | 0.00E+00 |
| Culex pipiens pipiens | 2 | 1.00E+07 | 24 | 14 | Pos | Neg | 2.12E+08 |
| Culex torrentium | 1 | 1.00E+06 | 24 | 14 | Neg | Neg | 0.00E+00 |
| Culex torrentium | 1 | 1.00E+06 | 24 | 14 | Neg | Neg | 0.00E+00 |
| Culex torrentium | 1 | 1.00E+06 | 24 | 14 | Neg | Neg | 0.00E+00 |
| Culex torrentium | 1 | 1.00E+06 | 24 | 14 | Neg | Neg | 0.00E+00 |
| Culex torrentium | 1 | 1.00E+05 | 24 | 14 | Neg | Neg | 0.00E+00 |
| Culex torrentium | 1 | 1.00E+05 | 24 | 14 | Neg | Neg | 0.00E+00 |
| Culex torrentium | 1 | 1.00E+05 | 24 | 14 | Neg | Neg | 0.00E+00 |
| Culex torrentium | 1 | 1.00E+05 | 24 | 14 | Neg | Neg | 0.00E+00 |
| Culex torrentium | 1 | 1.00E+05 | 24 | 14 | Neg | Neg | 0.00E+00 |
| Culex pipiens pipiens | 2 | 1.00E+07 | 24 | 14 | Pos | Pos | 2.55E+08 |
| Culex torrentium | 1 | 1.00E+05 | 24 | 14 | Neg | Neg | 0.00E+00 |
| Culex torrentium | 1 | 1.00E+05 | 24 | 14 | Neg | Neg | 0.00E+00 |
| Culex torrentium | 1 | 1.00E+05 | 24 | 14 | Neg | Neg | 0.00E+00 |
| Culex torrentium | 2 | 1.00E+07 | 24 | 14 | Pos | Pos | 2.66E+08 |
| Culex pipiens pipiens | 2 | 1.00E+05 | 24 | 14 | Pos | Neg | 2.87E+08 |
| Culex torrentium | 1 | 1.00E+05 | 24 | 14 | Neg | Neg | 0.00E+00 |
| Culex torrentium | 1 | 1.00E+05 | 24 | 14 | Neg | Neg | 0.00E+00 |
| Culex torrentium | 1 | 1.00E+05 | 24 | 14 | Neg | Neg | 0.00E+00 |
| Culex torrentium | 1 | 1.00E+05 | 24 | 14 | Neg | Neg | 0.00E+00 |
| Culex pipiens pipiens | 1 | 1.00E+06 | 24 | 14 | Neg | Neg | 0.00E+00 |
| Culex pipiens pipiens | 1 | 1.00E+06 | 24 | 14 | Neg | Neg | 0.00E+00 |
| Culex pipiens pipiens | 1 | 1.00E+06 | 24 | 14 | Neg | Neg | 0.00E+00 |
| Culex torrentium | 2 | 1.00E+06 | 24 | 14 | Pos | Neg | 3.11E+08 |
| Culex pipiens pipiens | 1 | 1.00E+06 | 24 | 14 | Neg | Neg | 0.00E+00 |
| Culex pipiens pipiens | 1 | 1.00E+06 | 24 | 14 | Neg | Neg | 0.00E+00 |
| Culex pipiens pipiens | 1 | 1.00E+06 | 24 | 14 | Neg | Neg | 0.00E+00 |
| Culex pipiens pipiens | 1 | 1.00E+06 | 24 | 14 | Neg | Neg | 0.00E+00 |
| Culex pipiens pipiens | 1 | 1.00E+06 | 24 | 14 | Neg | Neg | 0.00E+00 |
| Culex pipiens pipiens | 1 | 1.00E+06 | 24 | 14 | Neg | Neg | 0.00E+00 |
| Culex torrentium | 1 | 1.00E+07 | 24 | 14 | Pos | Pos | 3.13E+08 |
| Culex pipiens pipiens | 1 | 1.00E+06 | 24 | 14 | Neg | Neg | 0.00E+00 |
| Culex pipiens pipiens | 1 | 1.00E+06 | 24 | 14 | Neg | Neg | 0.00E+00 |
| Culex pipiens pipiens | 1 | 1.00E+06 | 24 | 14 | Neg | Neg | 0.00E+00 |
| Culex pipiens pipiens | 1 | 1.00E+07 | 24 | 14 | Neg | Neg | 0.00E+00 |
| Aedes albopictus | 2 | 1.00E+06 | 24 | 14 | Pos | Pos | 3.67E+08 |
| Culex torrentium | 2 | 1.00E+06 | 24 | 14 | Pos | Neg | 3.76E+08 |
| Culex pipiens pipiens | 1 | 1.00E+07 | 24 | 14 | Neg | Neg | 0.00E+00 |
| Culex torrentium | 1 | 1.00E+06 | 24 | 14 | Pos | Neg | 3.94E+08 |
| Culex pipiens pipiens | 1 | 1.00E+07 | 24 | 14 | Neg | Neg | 0.00E+00 |
| Culex pipiens pipiens | 1 | 1.00E+07 | 24 | 14 | Neg | Neg | 0.00E+00 |
| Culex pipiens pipiens | 2 | 1.00E+07 | 24 | 14 | Pos | Neg | 4.46E+08 |
| Culex pipiens pipiens | 1 | 1.00E+07 | 24 | 14 | Neg | Neg | 0.00E+00 |
| Culex pipiens pipiens | 1 | 1.00E+07 | 24 | 14 | Neg | Neg | 0.00E+00 |
| Culex pipiens pipiens | 1 | 1.00E+07 | 24 | 14 | Neg | Neg | 0.00E+00 |
| Culex pipiens pipiens | 1 | 1.00E+07 | 24 | 14 | Neg | Neg | 0.00E+00 |
| Culex pipiens pipiens | 1 | 1.00E+07 | 24 | 14 | Neg | Neg | 0.00E+00 |
| Culex pipiens pipiens | 1 | 1.00E+07 | 24 | 14 | Neg | Neg | 0.00E+00 |
| Culex pipiens pipiens | 1 | 1.00E+07 | 24 | 14 | Pos | Pos | 4.64E+08 |
| Culex pipiens pipiens | 1 | 1.00E+07 | 24 | 14 | Neg | Neg | 0.00E+00 |
| Culex pipiens pipiens | 1 | 1.00E+07 | 24 | 14 | Neg | Neg | 0.00E+00 |
| Culex pipiens pipiens | 1 | 1.00E+07 | 24 | 14 | Neg | Neg | 0.00E+00 |
| Culex pipiens pipiens | 1 | 1.00E+05 | 24 | 14 | Neg | Neg | 0.00E+00 |
| Culex pipiens pipiens | 1 | 1.00E+05 | 24 | 14 | Neg | Neg | 0.00E+00 |
| Culex pipiens pipiens | 1 | 1.00E+05 | 24 | 14 | Neg | Neg | 0.00E+00 |
| Culex pipiens pipiens | 1 | 1.00E+05 | 24 | 14 | Neg | Neg | 0.00E+00 |
| Culex pipiens pipiens | 1 | 1.00E+05 | 24 | 14 | Neg | Neg | 0.00E+00 |
| Culex pipiens pipiens | 1 | 1.00E+05 | 24 | 14 | Neg | Neg | 0.00E+00 |
| Culex pipiens pipiens | 1 | 1.00E+05 | 24 | 14 | Neg | Neg | 0.00E+00 |
| Culex pipiens pipiens | 1 | 1.00E+05 | 24 | 14 | Neg | Neg | 0.00E+00 |
| Culex pipiens pipiens | 1 | 1.00E+05 | 24 | 14 | Neg | Neg | 0.00E+00 |
| Culex pipiens pipiens | 2 | 1.00E+05 | 24 | 14 | Pos | Pos | 5.04E+08 |
| Culex pipiens pipiens | 1 | 1.00E+05 | 24 | 14 | Neg | Neg | 0.00E+00 |
| Culex pipiens pipiens | 1 | 1.00E+05 | 24 | 14 | Neg | Neg | 0.00E+00 |
| Culex pipiens pipiens | 1 | 1.00E+05 | 24 | 14 | Neg | Neg | 0.00E+00 |
| Culex pipiens pipiens | 1 | 1.00E+05 | 24 | 14 | Neg | Neg | 0.00E+00 |
| Culex pipiens pipiens | 1 | 1.00E+05 | 24 | 14 | Neg | Neg | 0.00E+00 |
| Aedes albopictus | 2 | 1.00E+06 | 24 | 14 | Pos | Neg | 6.17E+08 |
| Culex pipiens pipiens | 1 | 1.00E+06 | 24 | 14 | Neg | Neg | 0.00E+00 |
| Culex pipiens pipiens | 1 | 1.00E+06 | 24 | 14 | Neg | Neg | 0.00E+00 |
| Culex pipiens pipiens | 1 | 1.00E+06 | 24 | 14 | Neg | Neg | 0.00E+00 |
| Culex pipiens pipiens | 1 | 1.00E+06 | 24 | 14 | Neg | Neg | 0.00E+00 |
| Culex pipiens pipiens | 1 | 1.00E+06 | 24 | 14 | Neg | Neg | 0.00E+00 |
| Culex pipiens pipiens | 1 | 1.00E+06 | 24 | 14 | Neg | Neg | 0.00E+00 |
| Culex pipiens pipiens | 1 | 1.00E+06 | 24 | 14 | Neg | Neg | 0.00E+00 |
| Culex pipiens pipiens | 1 | 1.00E+06 | 24 | 14 | Neg | Neg | 0.00E+00 |
| Culex pipiens pipiens | 1 | 1.00E+06 | 24 | 14 | Neg | Neg | 0.00E+00 |
| Culex pipiens pipiens | 1 | 1.00E+06 | 24 | 14 | Neg | Neg | 0.00E+00 |
| Culex pipiens pipiens | 1 | 1.00E+06 | 24 | 14 | Neg | Neg | 0.00E+00 |
| Culex pipiens pipiens | 1 | 1.00E+06 | 24 | 14 | Neg | Neg | 0.00E+00 |
| Culex torrentium | 2 | 1.00E+05 | 24 | 14 | Pos | Pos | 6.26E+08 |
| Culex pipiens pipiens | 2 | 1.00E+07 | 24 | 14 | Pos | Pos | 6.32E+08 |
| Culex pipiens pipiens | 1 | 1.00E+06 | 24 | 14 | Neg | Neg | 0.00E+00 |
| Culex pipiens pipiens | 1 | 1.00E+05 | 24 | 14 | Neg | Neg | 0.00E+00 |
| Culex pipiens pipiens | 1 | 1.00E+05 | 24 | 14 | Neg | Neg | 0.00E+00 |
| Culex pipiens pipiens | 1 | 1.00E+05 | 24 | 14 | Neg | Neg | 0.00E+00 |
| Culex pipiens pipiens | 1 | 1.00E+05 | 24 | 14 | Neg | Neg | 0.00E+00 |
| Culex pipiens pipiens | 1 | 1.00E+05 | 24 | 14 | Neg | Neg | 0.00E+00 |
| Culex torrentium | 2 | 1.00E+07 | 24 | 14 | Pos | Pos | 1.09E+09 |
| Culex pipiens pipiens | 1 | 1.00E+05 | 24 | 14 | Neg | Neg | 0.00E+00 |
| Culex pipiens pipiens | 1 | 1.00E+05 | 24 | 14 | Neg | Neg | 0.00E+00 |
| Culex pipiens pipiens | 1 | 1.00E+05 | 24 | 14 | Neg | Neg | 0.00E+00 |
| Culex pipiens pipiens | 1 | 1.00E+05 | 24 | 14 | Neg | Neg | 0.00E+00 |
| Culex pipiens pipiens | 1 | 1.00E+05 | 24 | 14 | Neg | Neg | 0.00E+00 |
| Culex torrentium | 2 | 1.00E+06 | 24 | 14 | Pos | Pos | 1.22E+09 |
| Culex pipiens pipiens | 1 | 1.00E+05 | 24 | 14 | Neg | Neg | 0.00E+00 |
| Culex pipiens pipiens | 1 | 1.00E+05 | 24 | 14 | Neg | Neg | 0.00E+00 |
| Culex pipiens pipiens | 1 | 1.00E+05 | 24 | 14 | Neg | Neg | 0.00E+00 |
| Culex torrentium | 2 | 1.00E+05 | 24 | 14 | Neg | Neg | 0.00E+00 |
| Culex torrentium | 2 | 1.00E+05 | 24 | 14 | Neg | Neg | 0.00E+00 |
| Culex torrentium | 2 | 1.00E+05 | 24 | 14 | Neg | Neg | 0.00E+00 |
| Aedes albopictus | 1 | 1.00E+06 | 24 | 14 | Neg | Neg | 0.00E+00 |
| Aedes albopictus | 1 | 1.00E+06 | 24 | 14 | Neg | Neg | 0.00E+00 |
| Aedes albopictus | 1 | 1.00E+06 | 24 | 14 | Neg | Neg | 0.00E+00 |
| Aedes albopictus | 1 | 1.00E+06 | 24 | 14 | Neg | Neg | 0.00E+00 |
| Culex torrentium | 2 | 1.00E+07 | 24 | 14 | Pos | Pos | 2.07E+09 |
| Aedes albopictus | 1 | 1.00E+06 | 24 | 14 | Neg | Neg | 0.00E+00 |
| Aedes albopictus | 1 | 1.00E+06 | 24 | 14 | Neg | Neg | 0.00E+00 |
| Aedes albopictus | 1 | 1.00E+06 | 24 | 14 | Neg | Neg | 0.00E+00 |
| Culex torrentium | 2 | 1.00E+07 | 24 | 14 | Pos | Neg | 2.65E+09 |
| Aedes albopictus | 2 | 1.00E+06 | 24 | 14 | Pos | Neg | 2.91E+09 |
| Culex pipiens pipiens | 2 | 1.00E+05 | 24 | 14 | Neg | Neg | 0.00E+00 |
| Aedes albopictus | 2 | 1.00E+07 | 24 | 14 | Pos | Pos | 3.51E+09 |
| Culex pipiens pipiens | 2 | 1.00E+05 | 24 | 14 | Neg | Neg | 0.00E+00 |
| Culex pipiens pipiens | 2 | 1.00E+05 | 24 | 14 | Neg | Neg | 0.00E+00 |
| Culex pipiens pipiens | 2 | 1.00E+05 | 24 | 14 | Neg | Neg | 0.00E+00 |
| Culex pipiens pipiens | 2 | 1.00E+05 | 24 | 14 | Neg | Neg | 0.00E+00 |
| Culex pipiens pipiens | 2 | 1.00E+05 | 24 | 14 | Neg | Neg | 0.00E+00 |
| Culex pipiens pipiens | 2 | 1.00E+05 | 24 | 14 | Neg | Neg | 0.00E+00 |
| Culex pipiens pipiens | 2 | 1.00E+05 | 24 | 14 | Neg | Neg | 0.00E+00 |
| Culex pipiens pipiens | 2 | 1.00E+05 | 24 | 14 | Neg | Neg | 0.00E+00 |
| Culex pipiens pipiens | 2 | 1.00E+05 | 24 | 14 | Neg | Neg | 0.00E+00 |
| Aedes albopictus | 2 | 1.00E+07 | 24 | 14 | Pos | Pos | 3.90E+09 |
| Culex pipiens pipiens | 2 | 1.00E+05 | 24 | 14 | Neg | Neg | 0.00E+00 |
| Culex pipiens pipiens | 2 | 1.00E+05 | 24 | 14 | Neg | Neg | 0.00E+00 |
| Culex pipiens pipiens | 2 | 1.00E+05 | 24 | 14 | Neg | Neg | 0.00E+00 |
| Culex pipiens pipiens | 2 | 1.00E+05 | 24 | 14 | Neg | Neg | 0.00E+00 |
| Culex pipiens pipiens | 2 | 1.00E+05 | 24 | 14 | Neg | Neg | 0.00E+00 |
| Culex pipiens pipiens | 2 | 1.00E+05 | 24 | 14 | Neg | Neg | 0.00E+00 |
| Culex pipiens pipiens | 2 | 1.00E+05 | 24 | 14 | Neg | Neg | 0.00E+00 |
| Culex pipiens pipiens | 2 | 1.00E+05 | 24 | 14 | Neg | Neg | 0.00E+00 |
| Culex pipiens pipiens | 2 | 1.00E+05 | 24 | 14 | Neg | Neg | 0.00E+00 |
| Aedes albopictus | 2 | 1.00E+07 | 24 | 14 | Pos | Pos | 4.77E+09 |
| Culex pipiens pipiens | 2 | 1.00E+05 | 24 | 14 | Neg | Neg | 0.00E+00 |
| Culex torrentium | 2 | 1.00E+07 | 24 | 14 | Pos | Pos | 5.04E+09 |
| Culex torrentium | 2 | 1.00E+07 | 24 | 14 | Pos | Pos | 5.18E+09 |
| Aedes albopictus | 2 | 1.00E+07 | 24 | 14 | Pos | Pos | 6.35E+09 |
| Culex pipiens pipiens | 2 | 1.00E+05 | 24 | 14 | Neg | Neg | 0.00E+00 |
| Culex pipiens pipiens | 2 | 1.00E+05 | 24 | 14 | Neg | Neg | 0.00E+00 |
